# Molecular docking analysis of zidovudine triphosphate with conserved residues from NS3 protein

**DOI:** 10.6026/973206300212550

**Published:** 2025-08-31

**Authors:** Ayesha Tazeen, Rafat Ali, Nida Jamil Khan, Anwar Ahmed, Shama Parveen

**Affiliations:** 1 Centre for Interdisciplinary Research in Basic Sciences, Jamia Millia Islamia, New Delhi, India; 2 Department of Biosciences, Jamia Millia Islamia, New Delhi, India; 3 Centre of Excellence in Biotechnology Research, College of Applied Medical Sciences, King Saud University, Riyadh, Saudi Arabia

**Keywords:** Dengue virus, NS3 protein, protein-ligand interaction, MD simulation

## Abstract

Dengue fever poses a significant global health concern, yet despite extensive research there is no specific antiviral therapy against the infection. Therefore, it is of interest to study the interaction between the highly conserved N-terminal of NS3 protein of DENV-4 and an FDA approved antiviral, zidovudine triphosphate (ZDV-TP), using molecular docking and molecular dynamics (MD) simulation studies. Docking showed significant binding between NS3 protein and ZDV-TP with -7.7 kcal/mol of binding energy. ZDV-TP interacted with the conserved and active site residues of N-terminal NS3 protease including Gly133, Thr134, Ser135, Gly151 and Asn152. These residues are crucial for viral replication and are highly conserved in all the serotypes of DENV. MD simulation studies showed that the NS3-ZDV-TP complex had no major conformational instability with an approximately linear trajectory up to 200 ns simulation. ZDV-TP was well anchored at its binding position and minimized the compactness as compared to NS3 protein only, indicating high affinity for NS3.

## Background:

Dengue viral infection has emerged as a major concern in the tropical and subtropical regions of the world. Several countries have become hyper-endemic for Dengue virus (DENV) due to the co-circulation of more than one antigenically different serotype, which thus warrants the need for effective drugs and vaccines [1]. The ~11 kb genome of DENV consists of a positive-sense ssRNA with a single ORF flanked by 5' and 3' untranslated regions (UTRs). The viral polyproteins are co- and post-translated into three structural (envelope, pre-membrane and capsid) and seven non-structural proteins (NS1, NS2A, NS2B, NS3, NS4A, NS4B and NS5). The NS proteins of DENV are expressed in the infected host cells where they participate in the replication and assembly of virion [2]. The ~ 69 kDa NS3 protein is a multifunctional protein having 618 amino acids. The N-terminal domain (180 amino acids) is a chymotrypsin-like serine protease consisting of six β-strands which form two β-barrels with the catalytic triad sandwiched between them. The C-terminal (181-618) exhibit helicase, ATPase and RTPase activities, which are essential for viral replication and capping [3, 4]. The NS3 protease domain requires the NS2B cofactor to form the NS2B-NS3 protease complex. It catalyzes the hydrolysis of peptide bonds using the Ser135 residue. Apart from enzymatic activities, the NS3 protein has also been reported to interact with several host factors [5, 6]. A study has shown that NS3 protein (both full length and isolated protease and helicase domain) and human host GAPDH interactions are involved in hepatic metabolic modifications which may lead to steatosis in Dengue infected patients [6].

NS2B-NS3 protease is associated with immune evasion by inhibiting type I interferon production in the human primary dendritic cells [7, 8-9]. Additionally, NS3 protein is a highly conserved (~70%) protein among all the serotypes of DENV, making it a promising target for screening drug candidates and assessing their efficacy [10]. With the recent advancement in the bioinformatics, initial screening of potential drugs has become much easier. Thus, in our present study we have analyzed the FDA approved antiviral drug against DENV to minimize the selection of inactive ligands. The drug Zidovudine (ZDV) is a di-deoxynucleoside molecule in which an azido group replaces the 3'-hydroxy group in the sugar moiety. The active form of ZDV is Zidovudine triphosphate (ZDV-TP) which is a nucleotide analogue of thymine and also prevents phosphodiester linkage formation required to complete the nucleic acid chains [11]. Therefore, it is of interest to study the interactions of ZDV-TP with NS3 protein for its potential to act as a promising antiviral against DENV.

## Methodology:

## Molecular docking:

Crystal structure of the NS3 protein of DENV-4 was retrieved from the RCSB PDB (5YW1) and visualized using PyMol (v1.7.4.5). NS2B and BPTI were removed and NS3 protein was modelled using online software Swiss Model for adding missing residues. Swiss-PDB Viewer (v4.1) was used for energy minimization of NS3 protein. Kollman United atom charges and polar hydrogens and were added to the NS3 protein using AutoDock tools v1.5.7. The grid centre point was set at X=56.674, Y=4.616 and Z=46.629 with box dimensions (Å) as X=64, Y=48 and Z=32, covering N-terminal residues of NS3 protein with exhaustiveness 8 using AutoDock tools. The three-dimensional structure of ZDV-TP with PubChem ID: 72187 were downloaded and energy was minimized using Pyrx software. Docking was performed using AutoDock Vina v1.2.5 and the final result was visualized using Discovery studio software (v4.5). In addition, the ligand efficiency was calculated using formula:

Ligand Efficiency (LE) = -ΔG/N

Where, LE is in kcal mol^-1^ non-H atom^-1^, N is number of non-hydrogen atoms present in the ligand and ΔG is binding affinity (kcal mol^-1^).

## Molecular dynamics (MD) simulation:

MD simulation was performed using GROMACS (v.2023) software and topology of ZDV-TP was defined using CHARMM General Force Field (CGenFF) program and cgenff_charmm2gmx_py3_nx2.py tools. The simulation was done using a blind cubic box of one nm distance at the edges containing simple point charge (SPC) water molecules with the chamrmm36-July2022 force field to completely cover NS3 protein and NS3-ZDV-TP complex in the solvent. Further, counter ions were added for neutralizing the system followed by minimization using the steepest descent step of 50000 steps. The simulation was performed with a temperature of 300 K and pressure of 1 bar for 200 ns under the NPT ensemble and final results were assessed using the tools available in the GROMACS package. Further, principal component analysis (PCA) was also evaluated to determine the decrease in dimensionality in large datasets. The principal components for NS3 protein and NS3-ZDV-TP complex were obtained by the solvation and diagonalization of eigenvalues and eigenvectors for covariance matrices and calculated using GROMACS tool gmx covar. Additionally, the binding energy was calculated for the NS3-ZDV-TP complex using gmx_MMGBSA amber based tool for the last 5000 frames.

## Results and Discussion:

The docking of NS3 protein (Figure 1a - see PDF) with ZDV-TP showed significant interactions with binding energy of -7.7 kcal/mol (Figure 1b - see PDF). Notably, the azido group (-N_3_) of ZDV-TP interacted with the functionally important active site residue (Ser135), Gly133 and Thr134 of the protease domain which suggests that ZDV-TP might have strong probability to act as NS3 protease inhibitor and might greatly reduce its activity. The active site residue, Ser135 also formed hydrogen bond with the phosphate group of ZDV-TP. Additionally, phosphate group of ZDV-TP also interacted with His51, the active site residue of protease catalytic triad and Asp129 by forming pi-anion/pi-cation bond. Further, ZDV-TP also interacted with Phe130 and Pro132 interacted by carbon-hydrogen bond. Lys131, Try150, Gly153, Val155 and Tyr161 interacted with ZDV-TP via Van der Waals interactions (Figure 1b - see PDF). The protease domain of NS3 protein of DENV consists of three key amino acids known as the catalytic triad (His51, Asp75 and Ser135) which facilitates the peptide bond cleavage. Ser135 acts as a nucleophile and attack carbonyl carbon of the peptide bond. His51 and Asp75 stabilizes the reaction intermediates [8, 12]. The nucleophilic attack of Ser135-O-nucleophile is produced by the His51 basic catalysis at position P1 of carbonyl group. It generates tetrahedral intermediates which is stabilized in oxyanion hole by hydrogen bond interactions with Gly153 residue of NS3 protease. The intermediate decomposes which results in the cleavage of the C-terminal and releases the amine segment. Moreover, the N-terminal segment, which is linked to NS3 protease through ester bond, is hydrolyzed by water molecules. Here, the His51 residue acts as base for increasing the nucleophilic nature of water molecules which releases the N-terminal segment by re-protonation of carboxylic acid followed by initiation of new catalytic cycle [8, 12]. Additionally, Gly133 and Thr134 are the adjacent residue of the catalytic triad and is one of the most conserved residues of NS3 protein of *Orthoflaviviruses*. Gly133 helps to generate the catalytically fit conformation or geometry of Ser135 and thus oxyanion hole. Whereas Thr134 presumably provide weak interactions with the Arg residue through hydrogen bond [13]. A study reported that substitutions of Gly133 and Thr134 with Ala residue in the NS3 protease of DENV resulted in a 10-fold and 2.2-fold decrease in catalytic efficiency, respectively [14]. ZDV-TP also formed hydrogen bond with Asn152 via hydrogen bonding, which is also located near the active site residues of the NS3 protease domain and is crucial for ligand interactions [13]. Salaemae *et al.* also showed that substitution of Asn152 with Ala residue resulted in significantly high 60-fold reduction of the NS3 protease catalytic efficiency. Moreover, Ala substitution of Gly151, which interacted ZDV-TP via Van der Waals interaction, completely inhibited NS3 protease activity [14]. The ligand efficiency (LE) helps to select a promising ligands by comparing average binding energy per atom values [15, 16]. The ligand ZDV-TP also showed significant LE of 0.36 kcal mol^-1^ non-H atom^-1^.

RMSD can be defined as root mean square distance or simply average distance between each pair of atoms of two different aligned biomolecules [17, 18]. The RMSD trajectory of NS3 protein was initially stable for upto 40 ns but fluctuated at different positions till 200 ns simulation. The might be due to the slightly flexible nature of the NS3 protein domains in solution for its functional implications [19]. The NS3-ZDV-TP complex did not show any major fluctuation with almost linear trajectory upto 200 ns simulation. There was an insignificant fluctuation at 75 ns and 175 ns only (Table 1 - see PDF and Figure 2a - see PDF). The average RMSD was 0.51 and 0.37 nm for the native NS3 protein and NS3-ZDV-TP complex, respectively. This indicates that the docked NS3-ZDV-TP complex remained in relatively stable structural conformation with minimum deviation throughout simulation as compared to NS3 protein only. RMSF determines the average deviation of a residue from its initial position over a period of time [18, 20]. The plot in Figure 2b (see PDF) shows that NS3-ZDV-TP complex residues has lower RMSF values as compared to the NS3 protein throughout the 200 ns simulation. The residues Gly133, Thr134, Ser135, Gly151 and Asn152 which interacted through hydrogen bond in the docking showed less fluctutions in the NS3-ZDV-TP complex as compared to the native protein (Figure 2b - see PDF). It implies that fluctuations in the NS3 protein were decreased due to reduced residual vibrations and less atomic movements after binding with ZDV-TP leading to decreased flexibility of the complex. SASA is the surface area of the exposed protein which can interact with the solvent due to its surface and electrostatic properties [21]. The average SASA value of NS3 protein remained fairly stable throughout 200 ns simulation whereas it slightly decreased for NS3-ZDV-TP complex after 50 ns but remained constant after that (Table 1 - see PDF and Figure 2c - see PDF). It signifies that the solvent accessible area of the NS3-ZDV-TP complex was slightly reduced as compared to the NS3 protein, which might be due to the localized conformational change in the exposed surface area of the NS3-ZDV-TP complex, hence complementing the docking results. The Rg determines the compactness, flexibility, overall size, shape and folding properties of the protein and ligand-bound protein complex [22]. The average Rg value of NS3 protein remained nearly stable throughout the 200 ns simulation, whereas it slightly decreased for the NS3-ZDV-TP complex after 50 ns (Table 1 - see PDF and Figure 2d - see PDF). It implies that the overall structure of the NS3 protein remained constant but was slightly altered upon ZDV-TP binding. Also, the decreased Rg value shows that the NS3-ZDV-TP complex might have a more rigid structure and is more compact after ZDV-TP binding.

PCA is a mathematical tool to analyze the essential collective atomic motion and large conformational changes in the receptor and ligand-bound receptor complex [23, 24]. The PCA plots of NS3 protein and NS3-ZDV-TP complex is illustrated in Figure 3a (see PDF) and 3b (see PDF). The eigenvalues and eigenvectors demonstrates the magnitude and direction of motion, respectively of NS3 protein and NS3-ZDV-TP complex. The plots in Figure 3a (see PDF) and 3b (see PDF) shows that the collective motion of NS3-ZDV-TP complex significantly decreased and occupies similar conformational space as NS3 protein. However, no overall switch in the motion of NS3 protein was observed after complex formation with ZDV-TP. The FEL analysis is based on the PCA and provides insight into the energy minima and conformational landscape of protein and protein-ligand complex in terms of energy and time. FEL can distinguish thermodynamic and kinetic properties between the protein and ligand bound protein complex [25]. FEL plots of the NS3 protein and NS3-ZDV-TP complex is shown in Figure 3c (see PDF) and 3d (see PDF). The dark blue region in the FEL plot indicates energy minima for lower energy or stable state of the NS3 and NS3-ZDV-TP complex. It shows the energetically favored and thermodynamically stable conformational state of the NS3 protein and NS3-ZDV-TP complex. Hydrogen bonding determines stability of the polar interaction between the receptor protein and ligand [26]. The analysis showed that there was a slight change in number of hydrogen bond in the NS3 protein upon ZDV-TP binding and complex formation (Figure 4a-b - see PDF and Table 1 - see PDF). In addition, the probability distribution function (PDF) plots were also calculated, which also showed a slight change in the intramolecular hydrogen bonds but overall, there was good consistency. It signifies that upon binding, ZDV-TP might have slightly altered NS3 protein conformations. The secondary structure content of the NS3 protein and NS3-ZDV-TP complex was assessed as a function of time. Secondary structure assignments including β-sheet, α-helix and turn in the NS3 protein and NS3-ZDV-TP complex were broken into individual residues. The analysis inferred that during the 200 ns simulation, the overall secondary structure contents of the NS3 and NS3-ZDV-TP complex were constant (Figure 4c-d - see PDF and Table 2 - see PDF). The percentage of β-sheets and coils was constant throughout simulation but there was a very minimal change in the α-helix percentage. The binding energy for the NS3-ZDV-TP complex was calculated using gmxMMGBSA amber based tool for the last 5000 frames where the RMSD of the NS3 protein and NS3-ZDV-TP complex showed less fluctuation. The change in the total Gibbs free energy for NS3-ZDV-TP complex was found to be -22 Kcal/ mol. It has beeen reported in various studies that gmxMMGBSA over estimates the binding energy for ligand and protein complexes and thus further experimental validation is necessary.

## Conclusion:

ZDV-TP interacted with conserved and active site residues of N-terminal NS3 protein which are essential for its enzymatic activities. These interacting residues are reported to be highly conserved among all the DENV serotypes and other members of *Orthoflaviviruses*. The MD simulation studies revealed that the complex formed by the NS3 protein and ZDV-TP is stable and compact. This data might help to further study the potential inhibitory effect of ZDV-TP against the NS3 protein of DENV in *in vitro* and *in vivo* setups.

## Funding:

None to declare
